# CD169^+^ Macrophages Capture and Dendritic Cells Instruct: The Interplay of the Gatekeeper and the General of the Immune System

**DOI:** 10.3389/fimmu.2018.02472

**Published:** 2018-10-26

**Authors:** Joanna Grabowska, Miguel A. Lopez-Venegas, Alsya J. Affandi, Joke M. M. den Haan

**Affiliations:** Department of Molecular Cell Biology and Immunology, Amsterdam University Medical Center, Cancer Center Amsterdam, Amsterdam Infection and Immunity Institute, Vrije Universiteit Amsterdam, Amsterdam, Netherlands

**Keywords:** CD169, siglec-1, sialoadhesin, macrophages, dendritic cells, T cell, antigen, cross-presentation

## Abstract

Since the seminal discovery of dendritic cells (DCs) by Steinman and Cohn in 1973, there has been an ongoing debate to what extent macrophages and DCs are related and perform different functions. The current view is that macrophages and DCs originate from different lineages and that only DCs have the capacity to initiate adaptive immunity. Nevertheless, as we will discuss in this review, lymphoid tissue resident CD169^+^ macrophages have been shown to act in concert with DCs to promote or suppress adaptive immune responses for pathogens and self-antigens, respectively. Accordingly, we propose a functional alliance between CD169^+^ macrophages and DCs in which a division of tasks is established. CD169^+^ macrophages are responsible for the capture of pathogens and are frequently the first cell type infected and thereby provide a confined source of antigen. Subsequently, cross-presenting DCs interact with these antigen-containing CD169^+^ macrophages, pick up antigens and activate T cells. The cross-priming of T cells by DCs is enhanced by the localized production of type I interferons (IFN-I) derived from CD169^+^ macrophages and plasmacytoid DCs (pDCs) that induces DC maturation. The interaction between CD169^+^ macrophages and DCs appears not only to be essential for immune responses against pathogens, but also plays a role in the induction of self-tolerance and immune responses against cancer. In this review we will discuss the studies that demonstrate the collaboration between CD169^+^ macrophages and DCs in adaptive immunity.

## Introduction

While the first recognized characteristic of macrophages was their excellent capacity to phagocytose, dendritic cells (DCs) were acknowledged for their superior ability to stimulate naïve T cell responses. However, ever since tissue macrophages and DCs showed overlapping expression of several markers and were both generated from monocytes in *in vitro* models, it has been debated whether these cell types were closely related and had equivalent functions. The introduction of unbiased single cell multi-parameter analyses on the protein and RNA level, and the generation of cell-type specific and inducible genetically modified mouse models has enabled a new understanding of the generation and functions of both macrophages and DCs, and has even led to a new nomenclature (1). The current view is that the two cell types have very different functions in the immune system. However, this viewpoint potentially overlooks functional collaborations between the two cell types. In this review we will focus on the interactions between lymphoid tissue resident CD169^+^ macrophages and DCs and how these support the activation of adaptive immune responses.

### DCs and macrophages are different cell types with different functions

The generation of macrophages is dependent on the growth factor M-CSF and occurs in three waves [reviewed by (2, 3)]. First, during early embryonic development, yolk sac-derived progenitors seed several peripheral tissues, such as the brain and the epidermis. A second wave of progenitors derive from the fetal liver and seed lungs and liver. These two types of macrophages are characterized by high expression of F4/80 and in general reconstitute autonomously. Additionally, they are thought to have a long half-life and exhibit local proliferation. After birth, monocytes develop from hematopoietic stem cells in the bone marrow and tissues, such as the intestines and the skin that continuously receive monocytes to generate macrophages. The latter macrophages generally express low levels of F4/80.

Macrophages form a very heterogeneous population of cells and their diversity in phenotype and function is a reflection of the variety of the tissues in which they reside [reviewed by (4, 5)]. They are best known for their capacity to phagocytose and eliminate pathogens and to alarm the immune system. In addition to this important function in immunosurveillance, they are essential for the clearance of apoptotic cells and suppression of (auto) immune responses and mediate resolution of inflammatory responses and tissue repair. Furthermore, depending on their tissue of residence, macrophages have important specialized functions in development, homeostasis and metabolism [discussed in more detail in (4, 6)]. The general view is that macrophages exert their functions locally in the tissues and that in steady state tissue resident macrophages do not migrate to secondary lymph nodes to activate naïve T cells. This latter function is attributed to DCs that also reside in tissues, but upon pathogen recognition, upregulate CCR7 and travel to the lymphoid organs. However, upon inflammation monocyte-derived macrophages or DCs may also acquire the capacity to travel to the lymph nodes and stimulate T cells, which is a matter that has to be further clarified (7).

Currently, three types of DCs are being recognized [reviewed by (8, 9)]. Conventional or classical DCs (cDCs) are continuously generated in the bone marrow and require Flt3L for their generation. Pre-cDCs seed the tissues and the lymphoid organs and have a half-life of 5–7 days. Upon activation and upregulation of CCR7, tissue cDCs migrate to the lymph nodes and can activate T cells. Within cDCs two subsets can be identified. The cDC1 is more specialized in the uptake of dying cells, cross-presentation and activation of CD8^+^ T cells, while cDC2 has a more important role in CD4^+^ T cell activation and B cell responses. The generation of these two subsets is dependent on different transcription factors. While cDC1 requires Batf3, Id2 and Irf8, cDC2 development depends on Irf4 and RelB and requires additional Notch2 and vitamin A signals (10). With regard to the surface phenotype, cDC1 can be identified by XCR1 and CLEC9A, and additionally by CD8α in lymphoid organs and by CD103 in peripheral tissues. On the other hand, Sirpα, CD11b and CD4 expression marks the cDC2 subset. Next to cDCs, pDCs form another class of DCs that also develop in a Flt3L-dependent manner. This lineage splits from the cDC lineage before the separation in cDC1 and cDC2. They can be identified by CD123, BDCA2, and BDCA4 in humans and by high expression of BST2 and Siglec-H and by low expression of CD11c and B220 in the mouse. Recent studies have indicated further heterogeneity in CD123-expressing pDCs (11, 12). While early studies indicate that pDCs can take up antigens and stimulate T cells upon activation, recent studies suggest that very pure pDC populations only produce IFN-I and are not able to activate T cells unless they are pre-treated with CD40L and IL-3 (13). This suggests a limited function for pDC in T cell activation.

Next to these two Flt3L-dependent DC subsets, DCs can differentiate from monocytes during inflammatory conditions (7). The function of these DCs in the regulation of adaptive immune responses remains to be elucidated.

### Antigen cross-presentation by macrophages and dendritic cells

Both macrophages and DCs process antigens via the classical endogenous and exogenous pathways and present these on their MHC class I and II molecules, respectively, but they differ in their capacity to cross-present exogenous antigens in MHC class I and to cross-prime CD8^+^ T cells. Cross-presentation was first described in 1976 by Bevan as the process in which CD8^+^ T cell responses were initiated against donor antigens restricted by recipient MHC molecules (14). This process is thought to be essential in the activation of anti-viral and anti-tumor specific CD8^+^ T cell responses. While a number of studies have shown that exogenous antigens can be cross-presented by different cell types including macrophages (15), the mouse cDC1 subset exhibits a higher capacity to cross-present and is especially equipped for the uptake of dead cells and the cross-presentation of cell-associated antigens (16–19). However, depending on the antigen and activation stimuli, mouse cDC2 and several human DC subsets are also able to cross-present (17, 20). There are two main routes of antigen processing exist that leads to cross-presentation. In the cytosolic route, antigens are transported from the endosomal/phagosomal pathway to the cytoplasm and this pathway depends on proteasomes and TAP. In the vacuolar route, antigens are degraded in the endosomal/phagosomal pathway and bind to recycling MHC class I molecules. This pathway relies on the activity of cathepsin S. DCs mainly utilize the cytosolic route, while macrophages and monocyte-derived DCs have been shown to use the vacuolar route of cross-presentation (21, 22). Recent studies have identified a number of molecules involved in vesicular trafficking that play a role in cross-presentation [see for more details reviews (15, 23, 24)]. One of the important factors for cross-presentation is the rate of antigen degradation. Macrophages are more proteolytically active than DCs, which impairs their capacity to cross-present (25). DCs prevent the acidification of their phagosomes and thereby inhibit proteolysis by the activity of NADPH oxidase 2 (NOX2) enzyme [reviewed in (26)]. The NOX2 enzyme may also contribute to the translocation of antigens to the cytosol by disrupting the phagosomal membrane. The longer preservation of antigens in DCs and stronger phagosome-cytotosol translocation compared to macrophages may be responsible for the more prominent role of DCs in cross-priming.

### Generation of CD169^+^ macrophages and their innate functions

Macrophages expressing high levels of CD169, also known as Siglec-1 or sialoadheasin, constitute a minor macrophage population present in lymphoid tissues (27, 28). While several macrophage populations in tissues have low levels of CD169, which can be upregulated upon exposure to IFN-I, this lymphoid resident population has a very high constitutive expression of CD169. CD169^+^ macrophages are situated on top of B cell follicles bordering the marginal sinus in the spleen and the subcapsular sinus (SCS) in the lymph nodes and are also known as metallophilic marginal zone macrophages and SCS macrophages, respectively. The presence of B cells is necessary for the generation of CD169^+^ macrophages, which is mediated by their production of LTα1β2 (29, 30). In addition, they require RANK, LXR, and M-CSF signals and their survival is further promoted by TNF-α (31–34). Currently it is unclear which precursor gives rise to CD169^+^ macrophages, although their low level of F4/80 expression would suggest that they are not derived from yolk sac precursors. After elimination, they are repopulated from monocytes (34).

The strategic position of CD169^+^ macrophages at the entry site of lymphoid tissues determines their function. CD169^+^ macrophages are the first cell type in the spleen and lymph nodes to bind particulate antigens and pathogens and they function as a filter to remove foreign particles from the lymph fluid and blood. When these cells are deleted by clodronate liposomes in an experimental setting, pathogens can disseminate to other organs as has been demonstrated for several viral, bacterial and parasitic infections (35–39). This particular observation coined the term “gatekeeper” to describe CD169^+^ macrophages. This first line of defense, capturing invading viruses and limiting their spread to other organs, is not only mediated via the physical binding and capture of pathogens. CD169^+^ macrophages also exert their protective functions by the production of cytokines, such as IFN-I, IL-1, and IL-18. This cytokine secretion not only prevents subsequent infection of other cells and activates innate lymphocytes that help to contain the early infection (40–42), but also acts on DCs and stimulate adaptive immune responses.

### Model systems to study CD169^+^ macrophages

Due to their low abundance and sensitivity to manipulation, CD169^+^ macrophages are quite an enigmatic and technically challenging subset to study. Although it is feasible to extract these cells from spleen or lymph nodes by combination of mechanistic dissociation and enzymatic digestion, they rapidly die and form apoptotic blebs that bind to interacting cells (41, 43, 44). This feature greatly hampers the purification of CD169^+^ macrophages using fluorescence-activated cell sorting (FACS) for *in vitro* analysis. Unfortunately, available *in vitro* models do not offer a satisfactory method to investigate this macrophage population. *In vitro* cultured macrophages can be treated with IFN-α, which induces CD169 expression on the cell surface, but it is not clear whether these cells exhibit other characteristics of the CD169^+^ macrophages present *in vivo*. Most studies investigating CD169^+^ macrophages take advantage of cell ablation tools, either chemical using clodronate liposomes or genetic using diphtheria toxin receptor (DTR) systems. Despite representing a very effective method for transient depletion of macrophages, clodronate liposomes lack specificity. This apoptosis-inducing agent is toxic for all phagocytosing cells including monocytes and DCs (45). Noteworthy, the treatment with clodronate liposomes affects the anatomy of the surrounding tissue and induces off-target effects on B cells (46). In comparison to clodronate liposomes, DTR-mediated cell ablation allows for conditional and targeted depletion of a cell subset engineered to express DTR. CD11c-DTR and CD169-DTR are two DTR transgenic mouse strains that deplete CD169^+^ macrophages (47, 48). Although the CD11c-DTR model mainly depletes cells with high expression of CD11c, thus DCs, it does not spare macrophages that express low levels of this DC marker (49). The CD169-DTR model, on the other hand provides a more specific approach to study CD169^+^ macrophages, leaving the DC population unaffected. The only other population affected by DT treatment in CD169-DTR model, are SIGN-R1^+^ marginal zone macrophages that express low levels of CD169 (47). Similarly, the LXR-α KO lack both CD169^+^ and SIGNR1^+^ splenic marginal zone macrophage subsets (34). More recently, CD169-Cre mice have been generated and when crossed to the ROSA26-YFP mice generate reporter mice (50). The CD169-Cre mice will allow the generation of CD169-specific conditional KO mice and is therefore expected to provide a wealth of new insights for this macrophage population.

## CD169^+^ macrophages and IFN-I production

Upon encounter with pathogens, such as viruses, CD169^+^ macrophages regulate pathogen spread and induce immune responses by producing IFN-I. IFN-I consist of a single IFN-β and several subtypes of IFN-α, that signal through IFN-I receptor, a shared receptor expressed in almost all cell types (51). The importance of IFN-I signaling is 2-fold: (1) IFN-I can induce intracellular antiviral responses to suppress viral replication in the infected cells (52), and (2) IFN-I can regulate both innate and adaptive immune responses that are required to clear pathogens. However, depending on the type of pathogen, the outcome of IFN-I actions can play both protective and detrimental roles to the host (53, 54).

### Viral infection of CD169^+^ macrophages results in IFN-I production

CD169^+^ macrophages rapidly produce IFN-I after infection and thereby restrict the spread of a variety of viruses including mouse cytomegaloviruses (CMV), herpesvirus, and lymphocytic choriomeningitis virus (LCMV) (55–58). Several studies using neurotropic vesicular stomatitis virus (VSV) infection show that IFN-I signaling is necessary for the survival of the mice. Upon VSV infection, IFN-I was shown to be largely produced by CD169^+^ macrophages and this prevented VSV from entering the central nervous system (38). Similarly, during experimental infection with recombinant modified vaccinia virus Ankara (MVA), CD169^+^ macrophages were found to be the main IFN-I producers (59). In this model, CD169^+^ macrophages recruited and activated NK cells upon MVA infection, which was dependent on the production of IFN-I by CD169^+^ macrophages. Additionally, MVA infection also induced inflammasome activation by CD169^+^ macrophages that led to pyroptotic cell death, cytokine burst, and recruitment of inflammatory cells (60).

### CD169^+^ macrophages recruit and prime IFN-I production by pDCs

Next to CD169^+^ macrophages, pDCs are well-known for their capacity to produce IFN-I. They express TLR7 and TLR9 and high basal levels of IRF7 that allows them to detect intracellular nucleic acids and to produce IFN-α immediately upon encounter with pathogens (61). pDCs are located mainly in the lymphoid organs, such as bone marrow, spleen, and lymph nodes, but not in non-lymphoid tissues. In steady state, pDCs can be found in the T cell zone and peri-follicular area of the lymph node. Upon infection with pathogens, such as VSV, pDCs migrate to the SCS and medulla, areas rich in CD169^+^ macrophages (38). pDCs were reported to account for half of the IFN-I produced upon VSV infection, which was dependent on the presence of CD169^+^ macrophages. In a another study, the migration of pDCs to SCS was shown to be mediated by CXCR3, chemokine receptor of CXCL9, CXCL10, amongst others (62). It was suggested that viral particles from the infected CD169^+^ macrophages could activate these migrating pDCs. However, the direct interaction between SCS CD169^+^ macrophages and pDCs and its consequences are still unclear.

In a malaria infection model, pDCs accounted for the majority of IFN-I produced which led to lethal outcomes of infected mice (63). Spaulding et al. reported that after infection with malaria, CD169^+^ macrophages sustained prolonged interaction with pDCs in the bone marrow and primed them to produce IFN-I. Thus, this study provides evidence of an active interaction between CD169^+^ macrophages and pDCs that may also occur in other lymphoid organs.

However, pDC-derived IFN-I may be dispensable in some situations. In a study that exploited an MCMV footpad infection model, pDC depletion using αBST2 antibodies led to an increase in MCMV escape from SCS and spread to other tissues (55). Nevertheless, this effect was moderate when compared to blocking IFN-I using anti-IFN-I receptor antibodies. In another MCMV model where MCMV was administered intraperitoneally, depletion of pDCs also resulted in an increase of viral spread and dissemination, but only when a low dose was used (64). pDCs were also demonstrated dispensable for survival of the mice upon infection with VSV and *Plasmodium* (38, 63). Thus, upon pathogen encounter by CD169^+^ macrophages, pDCs are recruited to amplify IFN-I signaling, however this is not always essential for pathogen clearance or mice survival. Nevertheless, pDC-derived IFN-I may still contribute to other aspects of immune responses.

### IFN-I augments cDCs to initiate adaptive immune responses

The initiation of adaptive immune response by cDCs involves multiple mechanisms including antigen presentation, co-stimulatory/inhibitory molecules, and immunomodulation by cytokines. Next to its role in inhibiting viral replication, IFN-I has been demonstrated to augment NK cell function, B cell isotype switching, and T cell survival and activation (65). IFN-I is also critical for the function of cDCs to fully activate naïve T cells as it stimulates the expression of co-stimulatory molecules, enhances responses to TLR-ligands and increases antigen presentation capacity (66–69). cDC1, in particular, require the presence of IFN-I for antigen cross-presentation and subsequent CD8^+^ T cell activation (70). Several reports have demonstrated IFN-activated cDC1 to be important for generating CD8^+^ T cell responses against tumor or viral infections (71–73). In fact, IFN-I signaling induced by viruses could enhance the development of CD8^+^ T cell-mediated anti-tumor responses as a vaccination strategy (70, 74, 75).

Studies have been performed to identify the source of IFN-I required for the maturation of cDCs. In a vaccination system using tumor protein antigen and an iNKT cell ligand α-GalCer, splenic pDCs produced high amounts of IFN-I (76). Importantly, prior to cDC1 trafficking to the white pulp for T cell stimulation, pDCs were found to cluster with cDC1s in the CD169^+^ macrophage-rich marginal zone and red pulp area of the spleen. It was further shown that abolishing IFN-I signaling in CD11c^+^ cells led to an impaired memory T cell formation. This was in line with a previous study, where pDCs were reported to promote the generation and survival of antigen-specific CD8^+^ T cells upon VSV infection (64). More recently, Brewitz and colleagues have demonstrated pDC-derived IFN-I to be important for CD8^+^ T cell activation by cDC1 when mice were exposed to MVA (62). After MVA infection, pDCs, cDC1s, and CD8^+^ T cells formed superclusters in the interfollicular area of the lymph node. This event was required for CD8^+^ T cell responses. Additionally, in a vaccination strategy using TLR7 agonist as an adjuvant, pDC-derived IFN-I was crucial for *in vivo* CD8^+^ T cell killing (77). These observations suggest an important cross-talk between IFN-I-producing pDCs and CD8^+^ XCR1^+^ cDC1 for an optimal CD8^+^ T cell activation in vaccination or viral infection.

The effect of IFN-I derived from CD169^+^ macrophages and pDCs on the function of cDCs is not limited to CD8^+^ T cell activation. Upon infection with *S. mansoni* eggs, IFN-I was needed for an optimal cDC activation, migration and induction of Th2 immune responses *in vivo* (78). In a DC-targeting vaccination using HIV gag-protein and poly(I:C) as an adjuvant, CD4^+^ Th1 responses were abolished upon interference with IFN-I signaling (79). Next to T cells, IFN-I signaling on DCs could also mediate B cell function including antibody production, isotype switching and the development of T follicular helper cells (80, 81). Thus, IFN-I stimulated DCs have an enhanced capacity to activate both humoral and cell-mediated adaptive immune responses.

A similar priming effect of IFN-I on cross-presentation has also been shown in human DCs (82, 83). In humans, the level of IFN-I is highly elevated and has been suggested to contribute to the break of tolerance in many autoimmune diseases (84). For example in systemic lupus erythematosus (SLE), the increased level of IFN-I produced by pDCs directly induced cDCs maturation and CD4^+^ T cell activation (85). In psoriasis, pDC-derived IFN-I was sufficient to drive T cells infiltration and psoriatic plaque lesion formation (86). Interestingly, the numbers of CD169-expressing monocytes/macrophages were increased in the circulation and affected tissues of patients with systemic sclerosis and multiple sclerosis (87, 88). More investigation is needed to clarify the intricate cross-talk of CD169^+^ macrophage and pDC-derived IFN-I, cDC1, and T cell immunity in human diseases.

### Suppressive effects of IFN-I

Of note, the role of IFN-I during an infection is largely context-dependent and can also result in immunosuppression. A sustained IFN-I production can lead to increase of IL-10 and a higher expression of PD-L1. In a model of a persistent infection using LCMV strain Docile, upregulation of PD-L1 expression by CD169^+^ macrophages was important to promote CD8^+^ T cell exhaustion and prevented lethal immunopathology (58). The increased expression of PD-L1 in CD169^+^ macrophages was also observed in infection model with other LCMV strains (89). In addition, chronic infection with LCMV led to a sustained IFN-I production that prevented mice from mounting immune responses to a secondary infection by VSV (90). This was due to a reduced viral replication in CD169^+^ macrophages and subsequent impaired antigen presentation and lack of adaptive immune responses, rather than immunosuppression. However, using a model of *E. coli*-induced septic shock and subsequent systemic challenge with ovalbumin (OVA)-containing viruses, Schwandt et al. demonstrated that mice with sepsis had reduced antigen-specific CD8^+^ T cell responses. This suppression was mediated by macrophage-derived IFN-I that hampered cDC1 function to activate CD8^+^ T cells (91). Together these studies indicate that during chronic infections IFN-I production by CD169^+^ macrophages inhibits activation of immune responses toward secondary infections.

In conclusion, the production of IFN-I by CD169^+^ macrophages, potentially amplified by pDC-derived IFN-I, can strongly stimulate cDC function and the activation of immune responses, but may also result in immunosuppression.

## CD169^+^ macrophages efficiently capture pathogens and mediate antigen transfer

Their strategic location in spleen and in lymph nodes endows CD169^+^ macrophages with the capacity to capture blood- and lymph-borne pathogens. In fact, CD169^+^ macrophages appear to be extremely efficient in this process, as showed by multiple groups using various infection models (37, 38, 40, 92–96). Having acquired viral antigens, CD169^+^ macrophages were reported to transfer antigen to DCs and B cells mainly contributing to the infection control but also to virus dissemination in some cases.

### CD169^+^ macrophages enable containment of viral infection and localized production of antigen

The role of CD169^+^ macrophages as efficient gatekeepers has been demonstrated in a large number of viral infections, such as adenovirus, vaccinia virus, West Nile virus, and VSV (37, 92, 97). Additionally, experiments with human immunodeficiency virus (HIV) and murine leukemia virus (MLV) models confirmed prompt and potent virus capture by these gatekeeping macrophages (93). Deletion of splenic CD169^+^ macrophages was reported to cause rapid dissemination of LCMV and herpes virus infection (35, 98). Along the same line, local depletion of SCS macrophages resulted in higher viral titers in the spleen and other organs providing direct evidence for the protective role of CD169^+^ macrophages in systemic viral spread (37, 38, 40) (99). This clearly demonstrated the importance of CD169^+^ macrophages in infection containment.

Paradoxically, CD169^+^ macrophages can also support virus replication (33, 38, 99). Enforced virus replication within CD169^+^ macrophages endowed them with the distinct feature of being a source of viral antigen that facilitated activation of adaptive immune responses. Accordingly, increased expression of inhibitory protein Usp18 rendered splenic CD169^+^ macrophages unresponsive to IFN-I. As a consequence, enhanced cytopathic VSV replication in these cells was facilitated (94). CD169^+^ macrophage-mediated VSV replication mediated a strong VSV-neutralizing antibody response that rescued infected animals. Positive correlation between viral replication in CD169^+^ macrophages and protective adaptive immune responses was also shown in LCMV infection (100).

### CD169^+^ macrophages transfer antigens to DCs in viral infections

Apart from effective viral capture and containment of the infection, CD169^+^ macrophages have been previously reported to directly present particulate antigens, immune complexes as well as intact virus particles to non-cognate and cognate B cells (37, 101–103). This process was shown to stimulate germinal center responses and production of high affinity antibodies (103, 104). While in these initial studies that used clodronate liposomes, B cells were still activated in the absence of SCS macrophages (38), a recent study indicated that absence of SCS macrophages led to defective B cell responses (105). This process of intact virus presentation to B cells by CD169^+^ macrophages has also been implicated in trans-infection of B cells, contributing to the virus dissemination rather than to the virus containment (discussed in more detail in section *CD169 as a viral receptor that mediates virus capture and trans-infection*).

Despite robust evidence proving the importance of CD169^+^ macrophages in the induction of anti-viral B cell responses, their role in the activation of T cell responses is still being elucidated. While a number of studies demonstrate that CD169^+^ macrophages are dispensable for T cell priming (35–38, 94, 97), interaction between CD169^+^ macrophages and cDC1s has been shown to promote anti-viral T cell responses (44, 106, 107). The study by Backer et al. indicated that CD169^+^ macrophages could transfer antigens to cDC1s for the stimulation of CTL responses (106). In line with this, Bernhard et al. showed that antigen transfer between CD169^+^ macrophages and cDCs also occurred in adenoviral infection. Interestingly, CD169^+^ macrophages were also able to directly present viral antigens to T cells bypassing the need for cDCs for T cell priming. While all epitopes, including low affinity peptides, were directly presented by CD169^+^ macrophages, cDC1s only cross-presented high affinity T cell epitopes (107).

Recently, the collaboration between CD169^+^ and cDC1s was investigated in more detail (44). This study revealed that the CD169 receptor enabled cell-cell contact with sialylated ligands on cDCs and thereby facilitated transfer of antigen to cDCs. In addition to mediating adhesion to DCs, CD169 has also been reported to support binding of innate-like lymphocytes and neutrophils (41, 108, 109). Remarkably, even upon disintegration, CD169^+^ SCS macrophage cell-derived blebs are able to bind to IL-17 lymphocytes and NK cells (41, 43). Apparently, CD169 acts as an adhesion receptor that facilitates the interaction of CD169^+^ macrophages with other innate immune cells.

Interestingly, *in vivo* blockade of CD169 receptor resulted in impaired MVA-specific, but not VSV-specific CD8^+^ T cell responses (44). This observation could be explained by the dispensability of the cross-presentation process during certain viral infections, such as VSV in which DCs are likely to be directly infected (94). Specifically, KLRG-1^low^ CD8^+^ T cells with memory potential were negatively affected upon CD169 blocking in MVA-infected animals, indicating that CD169^+^ macrophage-mediated antigen transfer to cDC1s might facilitate memory responses as well. In line with this, collaboration between splenic CD169^+^ macrophages and cDC1s was important for activation of memory CD8^+^ T cell responses in VSV infection (33).

Van Dinther et al. showed that CLEC9A/DNGR-1 expressed on cDC1 enhanced CD8^+^ T cell cross-priming of antigens targeted to CD169^+^ macrophages (44). CLEC9A/DNGR-1 binds to F-actin exposed on dying cells and while it does not increase antigen transfer, it enhances T cell responses toward cell-associated material and in viral infections (110–112). A number of studies have indicated the disappearance or death of CD169^+^ macrophages induced by viral infection or other inflammatory agents (44, 60, 105). This suggests that upon infection, CD169^+^ macrophages quickly die and thereby form a cellular substrate for antigen transfer by the cross-presenting cDC1. This process could be of particular importance in viral infections, such as MVA, that solely depend on cross-presentation as opposed to VSV where the virus directly infects DCs (44).

### CD169 as a viral receptor that mediates virus capture and trans-infection

A decade ago, CD169 expressed on monocyte-derived DCs was found to promote HIV infection. This discovery brought a paradigm shift in the HIV field with CD169 replacing DC-SIGN as the main capture receptor responsible not only for HIV adhesion, but also for trans-infection (113–116). Following binding of CD169 to virus membrane-associated glycolipids (GM3), HIV-1 and CD169 were demonstrated to travel together to and accumulate at a non-lysosomal compartment. Consequently, the concentration of HIV-1 and CD169 at the so called infectious synapse enabled trans-infection of CD4^+^ T cells (117). A similar trans-infection process was also shown to be important for henipavirus infection (118).

In a study that focused on MLV and HIV infection *in vivo*, CD169-mediated virus capture was also reported to occur via CD169 binding to gangliosides on the viral membrane (93). Interestingly, CD169^+^ macrophages that had captured MLV, but were not infected themselves, were responsible for trans-infection of permissive B cells which facilitated spread of the infection. Accordingly, considerably lower numbers of virus- infected cells were detected both in peripheral lymph nodes and spleen upon blocking of CD169 and in CD169-deficient mice. This clearly illustrated the importance of CD169 for effective virus dissemination. In line with this, MLV was also demonstrated to exploit CD169 expressed on primary mouse bone marrow macrophages for trans-infection of proliferating B cells (95). Apart from aforementioned retroviral models, a study performed in a porcine reproductive and respiratory syndrome virus (PRRSV) also experimentally addressed the role of CD169 in virus anchoring (119). The authors proved that the attachment of the virus was dependent on the sialic acid binding activity of the receptor that binds to sialylated viral glycoproteins on PRRSV.

While substantial evidence from retroviral studies validates CD169 as a viral receptor that is exploited by the pathogen for its dissemination, numerous studies in viral models have demonstrated the importance of CD169 expressing macrophages for the containment of viral infection and localized production of antigen. The latter suggests that these macrophages form a reservoir of viral antigen for transfer to cDC1. A small number of studies suggest that a similar process may take place in certain bacterial infections.

### CD169^+^ macrophages efficiently trap bacteria and allow trans-infection of cDCs

Similar to what has been shown in viral infections, several studies using the *Listeria monocytogenes* (Lm) model confirmed CD169^+^ macrophages as the initial cellular host that effectively traps the bacteria (36, 120–122). While as early as 2 h post-infection, the majority of Lm was detected within macrophages in the marginal zone, by 9 h CD11c^+^ DCs were the main cell type carrying Lm (121). Two photon microscopy results showed clustering of Lm-specific T cells that associated with CD11c^+^ DCs in periarteriolar lymphoid sheath (PALS), which was indicative of ongoing antigen presentation. At 24 h Lm-infection foci were mainly localized to PALS where Lm was shown to replicate extensively. Using a CD11c-DTR model that allows for CD11c depletion upon DT injection, the authors confirmed that Lm transport to the PALS and subsequent antigen presentation were dependent on the presence of cDCs. However, as mentioned already, CD11c-DTR model also abrogates CD11c- expressing CD169^+^ macrophages. Therefore, only subsequent experiments performed in the Batf3^−/−^ model, formally established the role of cross-presenting cDC1 in Lm delivery to the PALS (120, 122).

Recently, Perez et al. (122) also noted a shift in Lm distribution from CD169^+^ macrophages to cDC1 over the course of infection and showed that CD169^+^ macrophages mediate trans-infection of cDC1. Accordingly, while in wild type animals cDC1 formed clusters near Lm-infected CD169^+^ macrophages in the marginal zone and efficiently delivered Lm to PALS, in CD169-DTR mice such clusters were not present and transport to PALS was impaired. Therefore, the presence of CD169^+^ macrophages closely interacting with cDC1 promoted trans-infection and enabled subsequent Lm entry to the PALS.

Similar to viral infections, CD169^+^ macrophages also control the spread of bacteria. Perez and colleagues reported increased bacterial titers in the spleen and blood of CD169-DTR mice, suggesting that these macrophages impede Lm replication and prevent Lm dissemination (122). Finally, using a CD169-DTR-Batf3^−/−^ model, that allows for conditional depletion of CD169^+^ macrophages in cDC1-deficient mice, it was demonstrated that rapid Lm capture and clearance secured by CD169^+^ macrophages was instrumental for Lm control. Interestingly, the authors showed that cytosolic replication within CD169^+^ macrophages due to phagosomal escape was necessary for recruitment of cDC1.

While cDC1s have been identified as replication- permissive cellular hosts for Lm, a recent study demonstrated that CD169^+^ macrophages can have a similar role in pneumococcal septicaemia (123). Upon infecting CD169^+^ macrophages, *Streptococcus pneumoniae* evaded phagosomal clearance, proliferated intracellularly and after causing cell lysis disseminated to the bloodstream. The authors concluded that intracellular replication within CD169^+^ macrophages is crucial for resulting pneumococcal septicaemia.

Collectively, the findings from studies in bacterial infections, albeit almost exclusively performed in the Lm model, illustrate the importance of CD169^+^ macrophages as the initial cellular host. By capturing the bacteria, CD169^+^ macrophages initially mediate pathogen clearance and prevent systemic spread of the infection. However, they also serve as a bacterial reservoir that actually promotes propagation of the bacteria into the bloodstream at a later stage in the case of *Streptococcus pneumonia* or enable trans-infection of cDC1 by Lm. In addition to these two bacterial infections, the CD169 molecule has been shown to function as a receptor for bacterial uptake of pathogens rich in sialylated polysaccharides, such as *Neisseria meningitidis, Campylobacter jejuni*, and *Trypanosoma cruzi* (124–126). It remains to be established whether CD169^+^ macrophages function as a bacterial and/or antigen reservoir in these infections.

### Uptake and transfer of apoptotic cellular material by CD169^+^ macrophages and the implications for tolerance and cancer immunity

The distinction between self and non-self is essential for the proper function of the immune system. Next to their essential role in initiating immune responses specific for pathogens, CD169^+^ macrophages have also been shown to play a role in the induction of tolerance and anti-cancer immune responses.

### Role of CD169^+^ macrophages in tolerance

Continuous and non-inflammatory removal of apoptotic cell material is essential for the maintenance of tolerance. Using a transfer model of apoptotic cells, cDC1 cells were specifically shown to take up and present these cell-associated antigens to CD8^+^ T cells (16, 18, 19) and subsequently induce tolerance in the steady state (127). One of the first observations indicating a tolerogenic function for CD169^+^ macrophages was made by Miyake et al., who generated CD169-DTR mice in which all marginal zone macrophages were eliminated upon injection with DT (47). Upon injection of apoptotic cells loaded with a fragment of the myelin oligodendrocyte glycoprotein peptide (MOG peptide), an accumulation of apoptotic cell content was observed in the marginal zone in wild type mice, which prevented the development of EAE. Depletion of marginal zone macrophages via DT administration in CD169-DTR mice resulted in a failure of induction of tolerance and a switch in the uptake of apoptotic cells from CD8^+^ cDC1s to CD8^−^ cDC2s (47).

Next to cDC2s, also red pulp macrophages, have been accounted for the defective uptake of apoptotic cells and the abrogation of tolerance in the absence of marginal zone macrophages. When marginal zone macrophages were depleted by means of clodronate liposomes, an accumulation of apoptotic cells was detected in F4/80^+^ macrophages. This was correlated with the production of inflammatory cytokines and loss of tolerance induction (128).

In subsequent studies by McGaha et al. the interaction between CD169^+^ macrophages and DCs was investigated. In their system, intravenous injection of apoptotic cells induced the expression of CCL22 on CD169^+^ macrophages, which resulted in a coordinated clustering of CCR4-expressing cDC1s and regulatory T cells within the white pulp. The induction of tolerance was dependent on both CD169^+^ macrophages and CCR4 (129). In contrast, another study reported that CCL22 is produced by the cDC1s upon injection with apoptotic cells, showing that the role of the cell type that produces CCL22 remains to be clarified (130). However, together these studies indicate that marginal zone CD169^+^ macrophages and cDC1s are essential in the induction of tolerance via the uptake of apoptotic cells and suggest a functional collaboration in this process.

### Uptake of tumor cell material and exosomes by CD169^+^ macrophages stimulate anti-cancer immunity

The previously discussed role of CD169^+^ macrophages in mediating the removal of dying cells from the circulation to induce tolerance suggests that a similar process could potentially be involved in anti-tumor immunity. In this sense, a number of factors have been proposed to shift the balance of tolerance toward immunity. Whether DCs induce immunity is a context-dependent process, influenced by environmentally provided stimuli, stage and type of cell death as well as the location where it takes place (131, 132). An example of this has been provided by Lorenzi and colleagues, who demonstrated enhanced intracellular persistence of antigenic particles in cDC1 upon injection of tumor apoptotic cells in combination with IFN-I. After exposure to IFN-I, cDC1 not only contributed to the induction of OT-I proliferation, but also exhibited an enhanced lifespan and expression of co-stimulatory molecules (133). Since CD169^+^ macrophages can produce high amounts of IFN-I, in combination with antigen this could provide the optimal stimulus for DCs to be able to cross-present cell-associated tumor antigens and to induce T cell activation.

However, the question remains whether CD169^+^ macrophages have the capacity to cross-present tumor antigens autonomously. One of the first studies exploiting subcutaneously-injected dead cells showed these cells being transported throughout the lymphatic system to the lymph nodes, where SCS macrophages cross-presented dead cell-associated antigens to CD8^+^ T cells. Mice that were lacking SCS macrophages at the moment of vaccination did not reject the tumors successfully (134). Interestingly, in this model the CD169^+^ macrophages, and not cDC1, were thought to directly cross-prime CD8^+^ T cells. This is reminiscent of the direct presentation of adenoviral antigens in the study of Bernhard et al., although the latter cannot be formally referred to as cross-presentation (107). Further studies are necessary to determine whether CD169^+^ macrophages can cross-prime CD8^+^ T cells independently or always require the collaboration with cDC1s.

In a model in which apoptotic cells were injected *in vivo* and induced CD4^+^ T cell activation, again macrophages were shown to be the main cells involved in the uptake and in their absence or the absence of cDC1 the CD4^+^ T cell activation was significantly decreased (135). Of note, an exosomal pathway was indicated to play a role in the cell-associated antigen transfer of macrophages to DCs. Exosomes are produced by many cell types and consist of small membrane vesicles that contain proteins, lipids, and nucleic acids. These vesicles can mediate transfer of such encapsulated molecules and thereby facilitate communication between cells (136). Exosomes have been found to be efficiently taken up by CD169^+^ macrophages and cDC1 in the spleen (137). McLellan and colleagues demonstrated that exosomes can express high levels of α2,3-linked sialic acids and bind abundantly to CD169^+^ macrophages in the spleen. Interestingly, CD169-deficient mice raised stronger CD8^+^ T cell responses toward antigen-pulsed exosomes than wild type mice (138). A similar suppressive role of CD169^+^ macrophages was observed in the T cell response toward tumor-derived apoptotic vesicles (139).

These studies suggest that CD169^+^ macrophages scavenge exosomes and thereby prevent their uptake by other cell types. Proof of that concept was provided by Pittet and colleagues in a mice model bearing genetically modified B16F10 melanoma tumors. The authors observed that tumor-derived exosomes drained to the lymph node and bound to CD169^+^ macrophages, which prevented the interaction with B cells that produce tumor promoting IgG. Elimination of CD169^+^ macrophages by clodronate liposomes or by DT injection in the CD169-DTR mice promoted tumor growth. In the same study, melanoma-derived material was found in macrophages residing in the cancer-free sentinel lymph node of human biopsies, hinting to the potential relevance of these findings for human cancer research (140).

Several groups have reported association of the presence of CD169^+^ macrophages in lymph nodes with good tumor prognosis in human. Ohnishi and colleagues showed a correlation between CD169^+^ macrophages and CD8^+^ T cell infiltration in colorectal cancer, improving overall survival rates. Furthermore, they observed co-localization of CD8^+^ T cells and CD169^+^ macrophages in regional lymph node section stainings (141). Similarly, more recent work from the same group demonstrated that the presence of CD169^+^ macrophages in the lymph nodes was also correlated to CD8^+^ T cell infiltration in malignant melanoma, endometrial carcinoma (where higher numbers of NK cells were also found), breast cancer and bladder cancer (142–145), all leading to a better prognosis and increased survival rates. Quite remarkably, in the study in malignant melanoma, IFN-α producing cells were detected around CD169^+^ macrophages in the lymph node sinus area. Based on their morphology and marker expression, the authors hypothesized that the source of IFN-α, supporting the action of CD169^+^ macrophages, could be CD68^+^ macrophages and pDCs (142). Altogether, these studies present robust data illustrating the importance of CD169^+^ macrophages in lymph nodes in proficient anti-tumor responses, characterized by a consistent CD8^+^ T cell infiltration that benefits patient prognosis and survival. However, while CD169^+^ macrophages where shown to co-localize with CD8^+^ T cells, no direct evidence of antigen presentation by CD169^+^ macrophages was provided at a functional level. Therefore, there might be room for other more specialized immune cells, such as cDC1 to cooperate in the process of T cell priming.

### Vaccination strategies that target to CD169^+^ macrophages

The presence of CD169^+^ macrophages in lymph nodes draining different tumor types and their correlation with a better patient survival, their unique capacity to screen the lymphatic and blood circulation and, finally, their capacity to collaborate with DCs, all point to CD169^+^ macrophages as appealing targets for the design of anti-cancer vaccines. Until now, several vaccination strategies targeting CD169^+^ macrophages have been evaluated experimentally.

Due to their high specificity and the restricted expression pattern of CD169, monoclonal antibodies have been tested for antigen delivery to CD169^+^ macrophages. Upon anti-CD169-specific antibody targeting of OVA, strong CTL responses were generated mediated by antigen transfer to cDC1 (106). This effect was lost upon depletion of CD169^+^ macrophages by the administration of clodronate liposomes and was shown to be mediated by BATF3-dependent cDC1s (44). Antibody-mediated targeting of OVA to CD169^+^ macrophages also led to an isotype-switched and high affinity antibody production due to germinal center activity. CD169^+^ macrophages retained intact antigen on their surface for days and upregulated costimulatory molecules for B cell interaction upon activation (103). This feature of CD169^+^ macrophages to retain intact molecules on their membrane has been correlated with low expression of proteolytic enzymes (104).

On a different note, Delputte et al. demonstrated that monoclonal antibodies against CD169 were not only binding, but also being efficiently internalized in a clathrin-dependent manner. Immunotoxins or antigens could be delivered to CD169^+^ macrophages via antibody targeting, leading to killing of primary porcine macrophages and the generation of anti-HSA humoral responses, respectively (146, 147). It is not clear why certain studies report internalization and others long-term presence on the cell surface with antibody targeting. Both processes could occur simultaneously, but these divergent results could also be due to antibodies binding to different regions of the CD169 molecule.

In addition, liposomes have been used to target antigens to CD169^+^ macrophages. Chen and colleagues generated OVA-containing liposomes decorated with 3′-BPCNeuAc, a synthetic ligand of CD169, and showed that targeting of IFN-α stimulated bone marrow-derived mouse macrophages with 3′-BPCNeuAc-liposomes induced OVA specific T cell proliferation (148). Moreover, the same authors could also accomplish activation of iNKT cells by including the lipid antigen αGalCer in the 3′-BPCNeuAc-liposomes (149). CD169^+^ macrophages seem well equipped in stimulating NKT cells via CD1d, which subsequently help B cell responses (42, 150). Liposomes with the endogenous ligand for CD169, ganglioside GM3, have also been shown to bind to CD169^+^ monocyte-derived DCs (151). These studies indicate that also liposomal strategies could be employed to target antigens and activating agents to CD169^+^ macrophages.

## Concluding remarks

**Figure 1:**
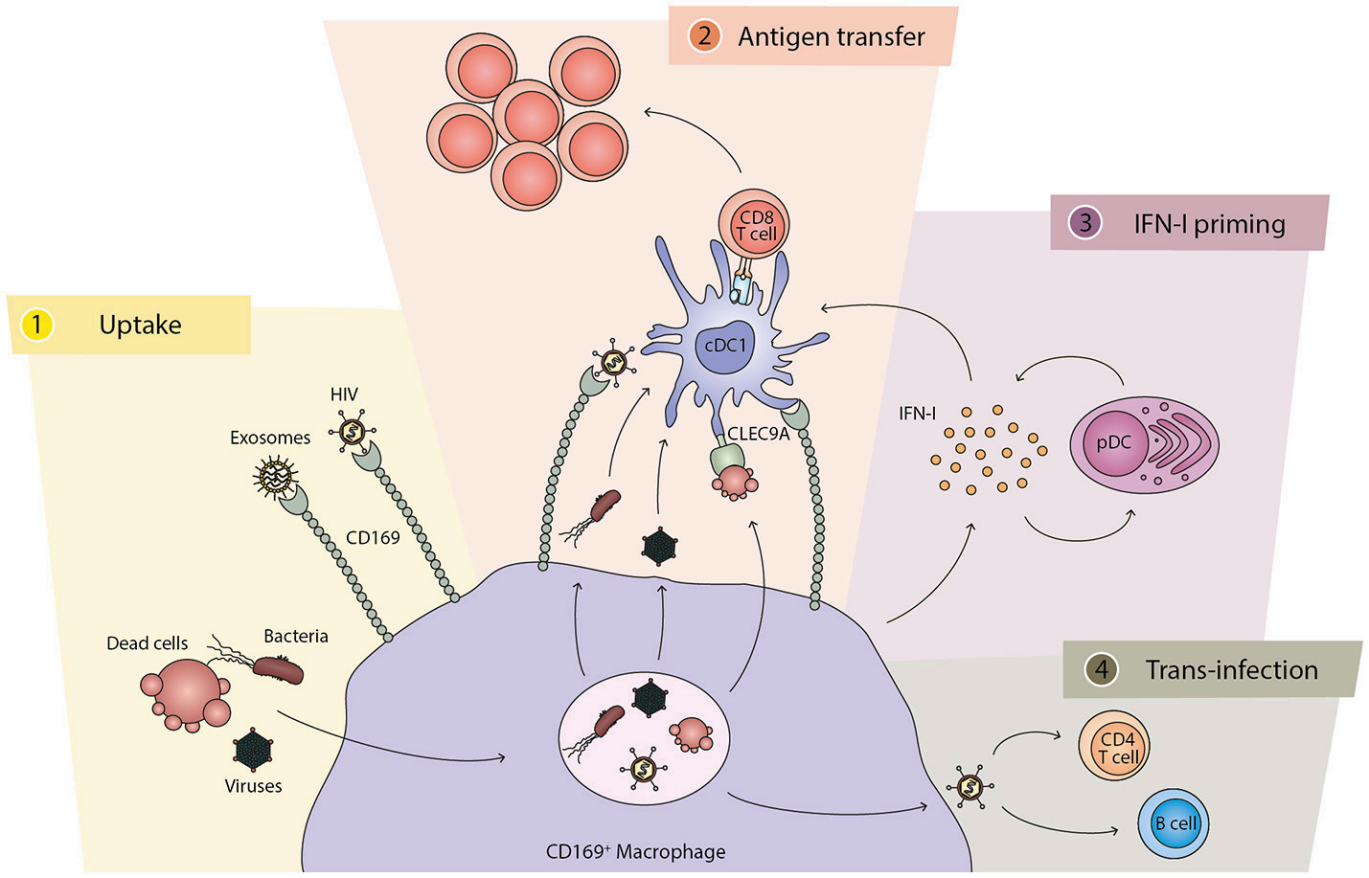
The different functions of CD169^+^ macrophages and their cross-talk with cDC1. (1) Uptake: CD169^+^ macrophages capture and phagocytose pathogens, including bacteria and viruses, as well as dead cells. The CD169 molecule also directly binds to exosomes and specific pathogens, such as HIV. (2) Antigen transfer: CD169^+^ macrophages directly interact and present antigens to cDC1s for the generation of antigen-specific CD8^+^ T cell responses. While HIV particles are transferred via CD169, other components of bacteria and viruses can be transferred to cDC1s from the macrophages. Dead cells can stimulate cDC1s via CLEC9A expressed on the cDC1. The interaction between CD169^+^ macrophages and cDC1s is dependent on binding of CD169 to sialic acid structures on cDC1s. (3) IFN-I priming: after encounter with bacteria, dead cells, or viruses, CD169^+^ macrophages secrete IFN-I that is required for optimal activation of cDC1s and T cells. Subsequently, pDCs are recruited and their IFN-I production further amplifies the signal. (4) Trans-infection: in the case of HIV and MLV, CD169^+^ macrophages can also mediate viral trans-infection to CD4 T cells and B cells.

In recent years a considerable number of studies have focused on the role that CD169^+^ macrophages play in the SCS of the lymph node and the marginal zone of the spleen (summarized in Figure 1). These studies, as discussed in this review, point to CD169^+^ macrophages as the main cell type to capture viruses, bacteria, dead cells and exosomes from the lymph fluid and the blood. This filtering capacity prevents further dissemination and enables a localized contained production of antigen that is efficiently transferred to DCs and B cells for the activation of adaptive immune responses. The collaboration between CD169^+^ macrophages and cDC1s is especially important in the activation of CD8^+^ T cell responses toward viral or tumor antigens. In this context, IFN-I derived from CD169^+^ macrophages and pDCs plays a crucial role for an appropriate cDC1 activation. However, a number of pathogens have exploited this pathway and utilize CD169^+^ macrophages as a niche to replicate and to mediate trans-infection of other cell types. In the coming years, the role of the human equivalent of this cell type will hopefully be elucidated and the development of treatment strategies to boost or down-regulate immune responses via the actions of the CD169^+^ macrophages may well be expected.

## Author contributions

All authors listed have made a substantial, direct and intellectual contribution to the work, and approved it for publication.

### Conflict of interest statement

The authors declare that the research was conducted in the absence of any commercial or financial relationships that could be construed as a potential conflict of interest.
